# Detection of SARS-CoV-2 RNA by In Situ Hybridization in Lung-Cancer Cells Metastatic to Brain and in Adjacent Brain Parenchyma

**DOI:** 10.3390/pathogens12060772

**Published:** 2023-05-29

**Authors:** Tibor Valyi-Nagy, Brian Fredericks, Jessica Wilson, Sajal Deea Shukla, Suman Setty, Konstantin V. Slavin, Klara Valyi-Nagy

**Affiliations:** 1 Department of Pathology, College of Medicine, University of Illinois at Chicago, Chicago, IL 60612, USA; bfrede3@uic.edu (B.F.); sshukla1@imsa.edu (S.D.S.); ssetty@uic.edu (S.S.); klaravn@uic.edu (K.V.-N.); 2 Department of Neurology, College of Medicine, University of Illinois at Chicago, Chicago, IL 60612, USA; jwilson004@gmail.com; 3 Illinois Mathematics and Science Academy, Aurora, IL 60506, USA; 4 Department of Neurosurgery, College of Medicine, University of Illinois at Chicago, Chicago, IL 60612, USA; kslavin@uic.edu; 5 Neurology Section, Jesse Brown Veterans Administration Medical Center, Chicago, IL 60612, USA

**Keywords:** SARS-CoV-2, brain, cancer, metastatic carcinoma, in situ hybridization, clinical case/report

## Abstract

The mechanisms by which severe acute respiratory syndrome coronavirus 2 (SARS-CoV-2) may spread to the human brain are poorly understood, and the infection of cancer cells in the brain by SARS-CoV-2 in Coronavirus disease 2019 (COVID-19) patients has been the subject of only one previous case report. Here, we report the detection of SARS-CoV-2 RNA by in situ hybridization in lung-cancer cells metastatic to the brain and adjacent brain parenchyma in a 63-year-old male patient with COVID-19. These findings suggest that metastatic tumors may transport the virus from other parts of the body to the brain or may break down the blood–brain barrier to allow for the virus to spread to the brain. These findings confirm and extend previous observations that cancer cells in the brain can become infected by SARS-CoV-2 in patients with COVID-19 and raise the possibility that SARS-CoV-2 can have a direct effect on cancer growth and outcome.

## 1. Introduction

Coronavirus disease 2019 (COVID-19) is caused by severe acute respiratory syndrome coronavirus 2 (SARS-CoV-2) [1,2,3]. The virus is highly transmissible and can easily be transmitted via mucous secretions, including respiratory droplets and saliva [1,4,5,6]. The initial site for viral infection is commonly the upper-respiratory system, with the virus, in more severe cases, spreading to and replicating in the lungs and evoking a strong immune response, potentially leading to cytokine storm syndrome, respiratory distress syndrome, respiratory failure, and death [2,3]. Severe infection may be associated with extrapulmonary disease, including cardiac, kidney, liver, gastrointestinal, and nervous-system injury [2]. Although SARS-CoV-2 RNA has been detected in multiple organs at low levels, the extent to which direct viral infection is responsible for extrapulmonary-tissue injury is unclear [2,7].

SARS-CoV-2 infection may cause a wide variety of neurologic sequelae that have predominately been attributed to systemic responses; however, there is evidence that SARS-CoV-2 is also neurotropic [7,8,9,10,11,12,13]. While immune activation and inflammation within the CNS have been proposed as primary drivers of neurologic disease in acute COVID-19, several studies of brain tissue from patients who died with acute COVID-19 also reported the detection of SARS-CoV-2 nucleic acid or the viral protein in the brain [7,9,11,12,13]. Recent observations suggest that the virus disseminates via an early viraemic phase, which seeds the virus throughout the body following infection of the respiratory tract in multiple anatomic sites, including throughout the brain, and that SARS-CoV-2 RNA may persist in the body for months (7). SARS-CoV-2 RNA and protein have been detected in situ in the brain stem, olfactory bulb, and cortex of post-mortem brain tissue from patients infected with SARS-CoV-2 [7,9,12,13]. The viral antigen was associated with the vascular compartment as well as with the prominent innate and adaptive immune activation but no widespread viral CNS infection was found [13]. A proposed central nervous system (CNS) entry mechanism of SARS-CoV-2 also includes olfactory transmucosal and transneural spread [8,9,10,11,12,13].

Studies have shown that COVID-19 causes complications, including the enhanced need for hospitalization and, overall, the increased risk of death in cancer patients, including those with lung cancer [14,15,16,17]. The angiotensin-converting enzyme 2 (ACE2) receptor—the primary mediator of SARS-CoV-2 entry into the cells—has shown to be expressed in several tumors, including lung-, colon-, and kidney-cancer tumors, and glioblastoma [18,19,20]. There are rare reports of the detection of SARS-CoV-2 in cancer tissues, including in colon-, renal-, and tongue-carcinoma tissues, and, in one case, in glioblastoma [19,20,21], suggesting that the direct virus infection of cancer cells may be a mechanism by which SARS-CoV-2 infection may have an impact on cancer pathogenesis. The SARS-CoV-2 infection of cancer cells in the brain has, to the best of our knowledge, been reported in only one publication: Lei et al. detected SARS-CoV-2 in surgically removed glioblastoma and the adjacent brain tissue from a convalescent patient of COVID-19 [20].

Here, we report the detection of SARS-CoV-2 RNA by in situ hybridization in lung-cancer cells metastatic to the brain and adjacent brain parenchyma in a 63-year-old male patient with COVID-19. These findings suggest that metastatic tumors may transport the virus from other parts of the body to the brain or may break down the blood–brain barrier to allow for the virus to spread to the brain. These findings confirm and extend previous observations that cancer cells in the brain can become infected by SARS-CoV-2 in patients with COVID-19 and raise the possibility that SARS-CoV-2 can have a direct effect on cancer growth and outcome.

## 2. Case Presentation

The patient was a 63-year-old African American male with a history of long-standing cigarette smoking (quit 6 months prior to presentation), chronic obstructive pulmonary disease (COPD), hypertension, gastropharyngeal reflux disease, anemia, and benign prostatic hyperplasia, who presented to an outside institution in April of 2020 with 5 days of right-sided weakness and gait instability. His history also included a 30 lb weight loss over the proceeding months, changes in the ability to read and calculate, as well as mild, subjective short-term memory complaints.

Magnetic resonance imaging (MRI) of the brain at the outside institution revealed two left-frontal lobe rim-enhancing lesions with central-diffusion restriction, measuring 6 × 5 mm and 8 × 13 mm on axial imaging, with extensive surrounding vasogenic edema and with a mild-mass effect on the lateral ventricle, and without mild-line shift. Computed tomography (CT) of the chest at the outside hospital showed filling defects in the pulmonary vasculature consistent with pulmonary emboli (PE), bilateral central-pulmonary consolidation, multiple spiculated nodular opacities (the largest being a 1.1 cm spiculated nodule on the right-upper lobe), diffuse-septal thickening, bilateral hilar-lymph-node enlargement, and mild prominence of the bilateral axillary lymph nodes. A CT scan of the abdomen with contrast revealed a necrotic left-adrenal gland with a large rim-enhancing mass of 2.1 × 2.5 × 3.2 cm. A bilateral lower-extremity duplex ultrasound revealed an acute left-lower extremity deep vein thrombosis (DVT). The patient declined inpatient biopsy and was discharged on oral dexamethasone for cerebral edema as well as on Lovenox and home oxygen for acute PE and left-lower-extremity DVT.

The patient followed up with outpatient neurosurgery at our institution 15 days later and was scheduled for planned admission for intravenous steroids and intracranial biopsy/resection with written patient consent.

**Figure 1:**
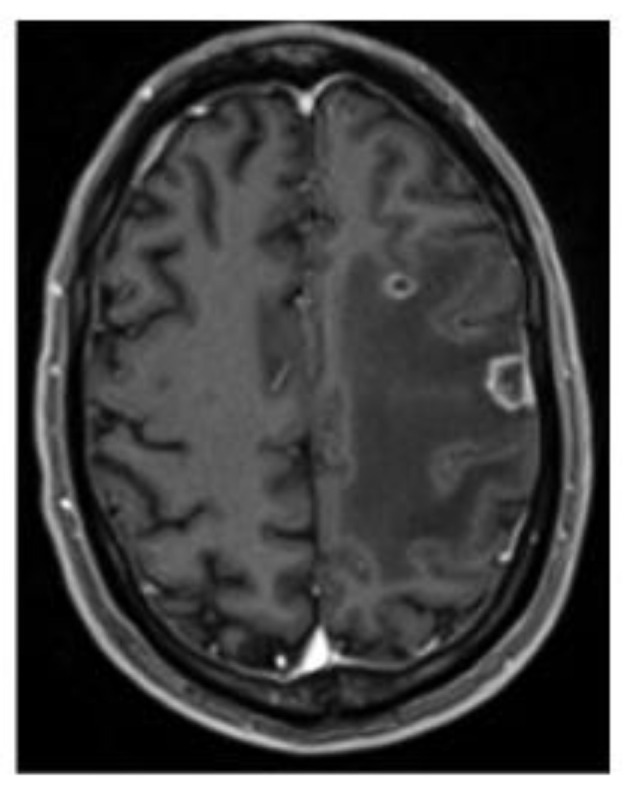
Brain axial T1 MRI with contrast, demonstrating two ring-enhancing lesions within the left-centrum semiovale with extensive adjacent edema.

Three days later, pre-admission SARS-CoV-2 testing by the ID NOW COVID-19 assay (Abbott Diagnostics Scarborough Inc) on a nasopharyngeal swab sample was positive. The ID NOW COVID-19 assay is a rapid molecular in vitro diagnostic test that uses isothermal nucleic-acid-amplification technology for the qualitative detection of nucleic acid from the SARS-CoV-2 virus. Admission proceeded as planned and he was afebrile with no oxygen requirements. The patient was transitioned to 4 mg of dexamethasone IV for 6 h and Lovenox was transitioned to heparin infusion. Pre-surgical MRI of the brain re-demonstrated the two left-frontal lobe rim-enhancing lesions with central-diffusion restriction, with mild progression of the surrounding vasogenic edema and a more pronounced mass effect, which then caused a 2 mm midline shift (Figure 1).

The imaging also showed two small regions of T2 FLAIR hyper intensities in the right-frontal lobe, one of which displayed possible diffusion restriction and a small region of contrast-enhancement along its lateral border. The largest left-frontal lobe lesion was resected through craniotomy. The intra-operative frozen section was consistent with metastatic carcinoma.

**Figure 2:**
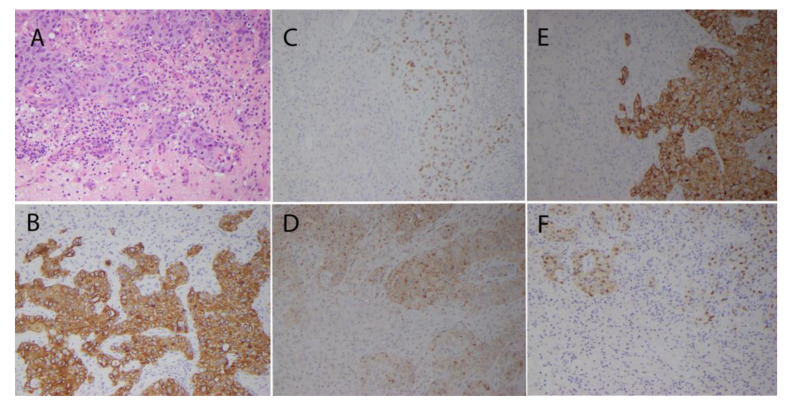
(**A**): hematoxylin- and eosin-stained section of resected metastatic carcinoma demonstrating large and cohesive tumor cells with prominent inflammation involving both tumor and adjacent brain tissue. (**B**): brown staining represents immunohistochemical detection of cytokeratin 7 in metastatic carcinoma cells. Brown staining represents immunohistochemical detection of TTF-1 (**C**), napsin A (**D**), cytokeratin 5/6 (**E**), and p63 (**F**) in metastatic carcinoma cells. Magnification is 400×.

Histopathologic examination of the resected tissue demonstrated metastatic carcinoma with histopathologic and immunohistochemical features consistent with adenosquamous carcinoma of lung origin (Figure 2).

The tumor cells were large and cohesive, and surrounded by small amounts of brain tissue (Figure 2A). There was predominantly chronic inflammation focally involving both the tumor and adjacent brain tissue (Figure 2A). Many tumor cells were positive for CAM5.2, cytokeratin 7 (Figure 2B), TTF-1, napsin A, cytokeratin 5/6, and p63 by immunohistochemistry, consistent with metastatic adenosquamous carcinoma of lung origin.

A CT scan of the chest/abdomen demonstrated an approximately 3 cm spiculated mass in the posterior segment of the right-upper lobe, suggestive of primary lung cancer. Associated satellite nodules were identified in the right lung as well as a left-lung nodule. There was bilateral-hilar lymphadenopathy, diffuse-septal thickening, as well as a left-adrenal mass.

The pathologic and clinical findings were consistent with non-small cell lung primary carcinoma T4N3M1c Stage IVB with metastases to the bilateral-hilar lymph nodes, lungs, adrenal glands, and brain. The patient was discharged in stable condition and was followed up with neurosurgery and oncology for treatment planning.

The patient was re-admitted 3 weeks after discharge for shortness of breath attributed to COPD exacerbation. SARS-CoV-2 testing by the ID NOW COVID-19 assay (Abbott Diagnostics Scarborough Inc) on a nasopharyngeal swab sample was positive. A CT scan of the chest demonstrated a mildly increased size of the right-upper lobe, a spiculated nodule and satellite nodules, new perihilar consolidations with increased interlobular septal-thickening, and ground-glass opacities, suggestive of worsening lymphangitic carcinomatosis with or without superimposed infection. While inpatient, the patient was started on treatment for acute COPD exacerbation with a 5-day course of 40 mg of prednisone and 750 mg of levofloxacin to finish the course for 5 days. The patient was weaned to room air and discharged as well as allowed to return home in stable condition. The patient presented to and expired at an outside hospital ten days after discharge from our institution. No autopsy was performed.

**Figure 3:**
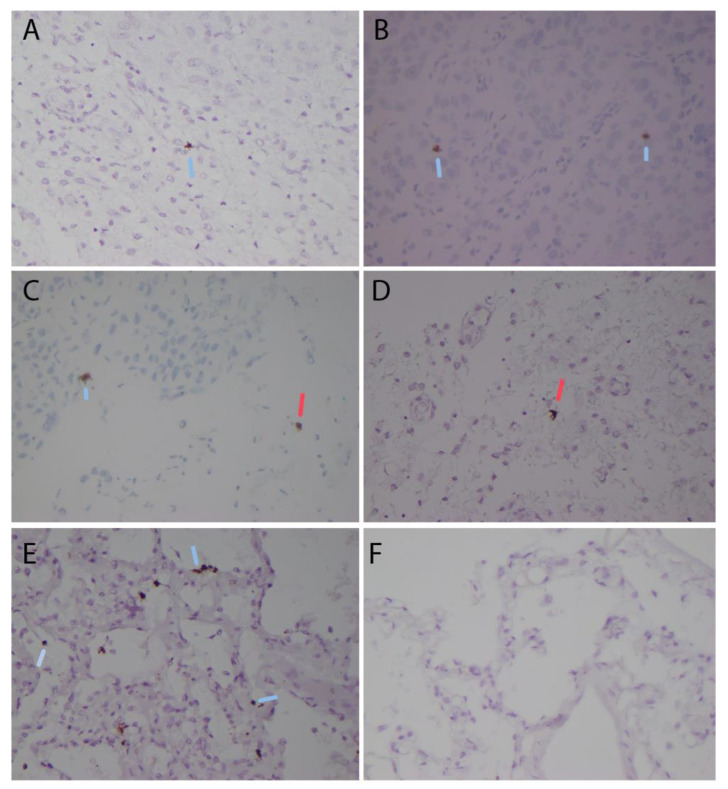
Detection of SARS-CoV-2 RNA by in situ hybridization (ISH) with the RNA Scope^®^ detection system and probe for genomic RNA coding for the spike protein in metastatic carcinoma. (**A**–**D**) patient tissue: brown staining represents SARS-CoV-2 RNA detected by in situ hybridization in scattered metastatic cancer cells (blue arrows) and in occasional cells with morphologic features of glial cells in adjacent brain tissue (red arrows). Positive and negative control tissues represent human-lung tissues with (**E**) and without (**F**) history of SARS-CoV-2 infection. Brown staining in (**E**) represents SARS-CoV-2 RNA detected by in situ hybridization in cells (blue arrows). Magnification is 400×.

A retrospective additional analysis of the lung-cancer tissue metastatic to the brain was performed with the use of a protocol approved by the Institutional Review Board of the University of Illinois in Chicago. Five microns of the tissue sections of the formalin-fixed paraffin-embedded tumor-tissue sections were examined for evidence of SARS-CoV-2 infection by in situ hybridization (ISH) with the RNA Scope^®^ detection system (RNAscope 2.5 assay kit with brown chromogenic dye, Advanced Cell Diagnostics, Inc., Newark, CA, USA, cat# 322300) and probe for genomic RNA coding for the spike protein (Advanced Cell Diagnostics, Inc., Newark, CA, USA, cat# 848561) per the manufacturer’s instructions and as reported by other studies [22,23,24]. SARS-CoV-2 RNA was detected by in situ hybridization in scattered metastatic cancer cells and in occasional cells with the morphologic features of glial cells in the adjacent brain tissue (Figure 3A through a 3D perspective). Positive and negative control tissues demonstrated appropriate staining (Figure 3E,F).

The number of viral RNA-positive cancer cells in five randomly selected 400× high-power microscopic fields was determined in two separate sections and ranged between 0 and 4 positive metastatic cancer cells per one high-power microscopic field, with an average of 1.5 positive viral RNA-positive cancer cells per one high-power microscopic field. The viral RNA-positive cancer cells represented up to 3% of the cancer cells in the most actively staining tumor areas based on the counting tissue areas with 100 cells.

The number of viral RNA-positive cells in the brain tissue adjacent to the metastatic cancer ranged between 0 and 2 positive cells per one high-power microscopic field, with an average of 0.4 viral RNA-positive cells per one high-power microscopic field. Viral RNA-positive cells represented up to 2% of the cells in the most actively staining brain-tissue areas based on counting 100 cells.

## 3. Discussion

Here, we reported the detection of SARS-CoV-2 RNA by in situ hybridization in lung-cancer cells metastatic to the brain and adjacent brain parenchyma in a 63-year-old male patient. There are rare reports of the detection of SARS-CoV-2 in cancer tissues, including in colon-, renal-, and tongue-carcinoma tissue, and, in one case, in glioblastoma [19,20,21]. SARS-CoV-2 infection of cancer cells in the brain has, to the best of our knowledge, been reported in only one publication, in which the authors detected SARS-CoV-2 in surgically removed glioblastoma and the adjacent brain tissue from a convalescent patient of COVID-19 [20]. The report indicates that SARS-CoV-2 may directly infect the cells of a primary brain tumor, of glioblastoma, and of the adjacent brain tissue; however, the mechanism of the virus infection of tumor cells and its significance to tumor pathogenesis are not evident from that case report.

The mechanisms by which SARS-CoV-2 reached the tumor cells and cells in the adjacent brain are also not evident from the analysis of our case of metastatic carcinoma. While the number of SARS-CoV-2-positive cells was small in both the metastatic tumor and adjacent brain areas, positivity was more frequent among tumor cells. Our studies do not provide an explanation for these findings. It seems possible that tumor cells became infected with SARS-CoV-2 in the lung and carried the virus to the brain, eventually leading to secondary infection of the brain parenchyma. Alternatively, our observations could be due to an enhanced affinity of SARS-CoV-2 for tumor cells over cells of the brain parenchyma upon the virus spreading to the brain through viremia or, less likely, through transneural spreading.

The clinical implications of these observations remain unknown but the detection of SARS-CoV-2 in metastatic lung-carcinoma cells raises the possibility that SARS-CoV-2 has the potential to directly modulate tumor pathogenesis. A limited number of viruses have been implicated in viral-mediated carcinogenesis in humans (i.e., Epstein–Barr virus, Kaposi’s sarcoma herpes virus, high-risk human papillomaviruses, Merkel cell polyomavirus, hepatitis B and C viruses, human immunodeficiency virus, type-1 and human T cell lymphotrophic virus, and type-1), and viruses are estimated to play an etiologic role in 10–15% of human-cancer cases globally [25,26]. Likewise, a subset of viruses are oncotropic and naturally possess onco-suppressive/oncolytic properties [27,28,29]. The effect, if any, of SARS-CoV-2 infection on the pathogenesis of the metastatic lung cancer of this patient cannot be assessed. Our findings, along with a limited number of other reports of SARS-CoV-2 infection in a variety of tumor types, suggest that additional studies are warranted to explore the potential direct effects of SARS-CoV-2 infection on human-cancer pathogenesis.

In summary, we report the detection of SARS-CoV-2 RNA by in situ hybridization in lung-cancer cells metastatic to the brain and adjacent brain parenchyma in a 63-year-old male patient with COVID-19. These findings raise the possibility that metastatic tumors may transport the virus from other parts of the body to the brain or may break down the blood–brain barrier to allow for the virus to spread to the brain. These findings confirm and extend previous observations that cancer cells can become infected by SARS-CoV-2 in patients with COVID-19 and provide additional information on possible mechanisms by which SARS-CoV-2 can have a direct effect on cancer growth and outcome.

## Data Availability

Data sharing not applicable.
